# A Vaccine Based on a Modified Vaccinia Virus Ankara Vector Expressing Zika Virus Structural Proteins Controls Zika Virus Replication in Mice

**DOI:** 10.1038/s41598-018-35724-6

**Published:** 2018-11-26

**Authors:** Patricia Pérez, María Q. Marín, Adrián Lázaro-Frías, Nereida Jiménez de Oya, Ana-Belén Blázquez, Estela Escribano-Romero, Carlos Óscar S. Sorzano, Javier Ortego, Juan-Carlos Saiz, Mariano Esteban, Miguel A. Martín-Acebes, Juan García-Arriaza

**Affiliations:** 1 Department of Molecular and Cellular Biology, Centro Nacional de Biotecnología (CNB), Consejo Superior de Investigaciones Científicas (CSIC), Madrid, Spain; 2 Department of Biotechnology, Instituto Nacional de Investigación y Tecnología Agraria y Alimentaria (INIA), Madrid, Spain; 3 Biocomputing Unit, Centro Nacional de Biotecnología (CNB), Consejo Superior de Investigaciones Científicas (CSIC), Madrid, Spain; 4 Centro de Investigación en Sanidad Animal, INIA-CISA, Valdeolmos, Madrid, Spain

## Abstract

Zika virus (ZIKV) is a re-emerging mosquito-borne flavivirus that affects humans and can cause severe neurological complications, including Guillain-Barré syndrome and microcephaly. Since 2007 there have been three large outbreaks; the last and larger spread in the Americas in 2015. Actually, ZIKV is circulating in the Americas, Southeast Asia, and the Pacific Islands, and represents a potential pandemic threat. Given the rapid ZIKV dissemination and the severe neurological and teratogenic sequelae associated with ZIKV infection, the development of a safe and efficacious vaccine is critical. In this study, we have developed and characterized the immunogenicity and efficacy of a novel ZIKV vaccine based on the highly attenuated poxvirus vector modified vaccinia virus Ankara (MVA) expressing the ZIKV prM and E structural genes (termed MVA-ZIKV). MVA-ZIKV expressed efficiently the ZIKV structural proteins, assembled in virus-like particles (VLPs) and was genetically stable upon nine passages in cell culture. Immunization of mice with MVA-ZIKV elicited antibodies that were able to neutralize ZIKV and induced potent and polyfunctional ZIKV-specific CD8^+^ T cell responses that were mainly of an effector memory phenotype. Moreover, a single dose of MVA-ZIKV reduced significantly the viremia in susceptible immunocompromised mice challenged with live ZIKV. These findings support the use of MVA-ZIKV as a potential vaccine against ZIKV.

## Introduction

Zika virus (ZIKV) is a mosquito-borne virus from the family *Flaviviridae* and the genus *Flavivirus*^1^, closely related with other mosquito-borne viruses with public health importance such as Japanese encephalitis virus (JEV), West Nile virus (WNV), dengue virus (DENV), and yellow fever virus (YFV). The viral particle has 50 nm in diameter and contains an inner nucleocapsid composed of a linear plus-strand genomic RNA and multiple copies of the viral capsid (C) protein and an outer host cell-derived lipid bilayer bearing 180 copies each of two proteins: the viral membrane [M, a cleavage product of the premembrane (prM)] protein and the envelope (E) protein^2,3^. The open reading frame encodes a large polyprotein of 3,423 amino acids which is cleaved by viral and cellular proteases into 10 individual proteins: three structural proteins located at the N-terminal region that form the infectious virion (C, prM, and E), and seven non-structural proteins located at the C-terminal region involved in viral replication (NS1, NS2A, NS2B, NS3, NS4A, NS4B, and NS5)^2,3^. ZIKV is transmitted to humans primarily through the bite of infected mosquitoes from genus *Aedes*, mainly by *A. albopictus* and *A. aegypti*, both widely distributed throughout the tropical and subtropical regions of the world, with the habitat of *A. albopictus* extending further into cool temperate regions^2,3^. Furthermore, ZIKV can also be transmitted from mother to child during pregnancy or spread through sexual contact, breastfeeding, or blood transfusion^2,3^. The multiple modes of ZIKV transmission make it difficult to develop control strategies against the pathogen.

ZIKV was discovered in Uganda in 1947, but was confined for the first 60 years to an equatorial zone across Africa and Asia^2,3^. However, in 2007 a ZIKV outbreak emerged in Yap Island, in the Western Pacific Ocean, and between 2013 to 2014 a second larger outbreak spread eastward to French Polynesia and other Pacific Islands that finally reached Latin America in 2015, and disseminated further to North America in 2016; as a consequence, the World Health Organization (WHO) declared the Public Health Emergency of International Concern in February 2016^2,3^. Actually, ZIKV is circulating in the Americas, Southeast Asia, and the Pacific Islands, and represents a potential pandemic threat^2,3^. In addition, since early 2015, there have been an increasing number of travel-related imported ZIKV cases in non-endemic countries and it is predicted that a large portion of the tropical and sub-tropical regions of the globe will have suitable environmental conditions for ZIKV mosquito transmission. Thus, there is currently a high risk of introducing and establishing new autochthonous transmission in these areas^2,3^.

In most cases ZIKV infection causes no symptoms or only a mild self-limiting illness, but recent epidemiological studies derived from outbreaks in 2007 and 2015 to 2016 linked ZIKV infection to a rising number of concerning severe neurological diseases, including Guillain-Barré syndrome and microcephaly^2,3^. Thus, the development of a safe and efficacious vaccine against ZIKV is critical given the rapid dissemination of the virus and the severe neurological and teratogenic sequelae associated with ZIKV infection. There are currently vaccine candidates in phase I or II clinical trials, and others under development^4,5^. These vaccine candidates include various technologies and approaches, such as inactivated ZIKV, recombinant viral vectors, DNA plasmid vaccines, mRNA-based vaccines, and peptide-based vaccines^4,5^. Zika vaccine development is mainly based on the whole inactivated organism or in vectored expression of prM and E structural proteins, as occurred with other flaviviral vaccines like JEV and DENV.

The highly attenuated poxvirus modified vaccinia virus Ankara (MVA) has been extensively used in numerous preclinical and clinical trials as a vaccine vector against several infectious diseases, being a cost-effective, safe and efficacious vector^6,7^. In addition, recombinant MVA vaccines express high levels of the heterologous antigens, and are potently immunogenic inducing antigen-specific humoral and T cellular immune responses^6,7^. Therefore, MVA should be a potential good vector to develop a vaccine against ZIKV.

Here, we have developed an MVA-based vaccine candidate (termed MVA-ZIKV) expressing the ZIKV prM and E structural proteins, and have characterized: (i) *in vitro*, expression of ZIKV proteins in infected cells, production of virus-like particles (VLPs) and genetic stability of the vector in cell culture at low multiplicity of virus infection; (ii) *in vivo*, in immunized mice, ability to activate B- and T-cell responses through the production of ZIKV neutralizing antibodies in serum, and induction of ZIKV-specific CD8^+^ T cells in splenocytes; and (iii) vaccine efficacy after live ZIKV challenge in susceptible immunocompromised mice. The results showed that MVA-ZIKV produced VLPs, is genetically stable and highly immunogenic in mice, activating B- and T-cell immune responses with the induction of good titers of neutralizing antibodies against ZIKV, together with potent and polyfunctional ZIKV-specific CD8^+^ T cell responses. Interestingly, a single dose of the MVA-ZIKV vaccine can control ZIKV replication in susceptible immunocompromised mice challenged with ZIKV.

## Results

### Generation and *in vitro* characterization of MVA-ZIKV

**Figure 1:**
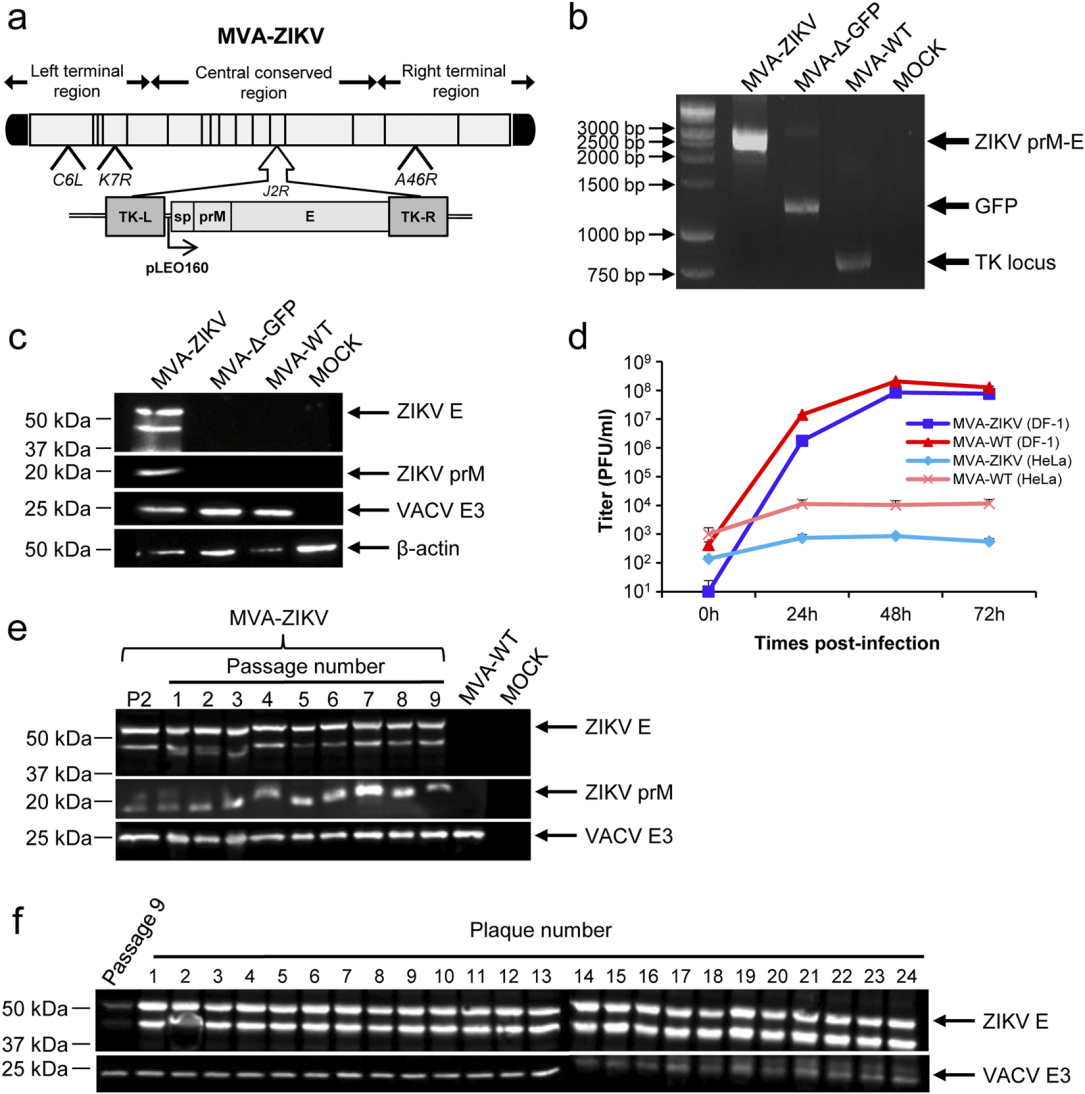
Generation and *in vitro* characterization of MVA-ZIKV. **(a)** Scheme of the MVA-ZIKV genome map. The ZIKV signal peptide (sp) following by the ZIKV prM-E structural genes (isolate Z1106033) are driven by the novel VACV synthetic pLEO160 promoter and are inserted within the VACV TK viral locus (J2R). The deleted VACV *C6L*, *K7R*, and *A46R* genes are indicated. TK-L, TK left; TK-R, TK right. **(b)** PCR analysis of the VACV TK locus. Viral DNA was extracted from DF-1 cells mock infected or infected at 5 PFU/cell with MVA-ZIKV, MVA-Δ-GFP, or MVA-WT. Primers spanning the TK locus-flanking regions were used for PCR analysis of the ZIKV genes inserted within the TK locus. DNA products are indicated by an arrow on the right. A molecular size marker (1-kb ladder) with the corresponding sizes (base pairs) is indicated on the left. **(c)** Expression of ZIKV prM and E proteins. DF-1 cells were mock infected or infected at 5 PFU/cell with MVA-ZIKV, MVA-Δ-GFP, or MVA-WT. At 24 hpi, cells were lysed, fractionated by 10% SDS-PAGE, and analyzed by Western blotting. Arrows on the right indicate the positions of the ZIKV prM and E proteins, the VACV E3 protein or β-actin. The sizes of standards (in kDa) are indicated on the left. **(d)** Viral growth kinetics of MVA-ZIKV. Monolayers of permissive DF-1 or non-permissive HeLa cells were infected at 0.01 PFU/cell with MVA-WT or MVA-ZIKV. At different times postinfection (0, 24, 48, and 72 hpi), virus titers in cell lysates were quantified by a plaque immunostaining assay. The means of results from two independent experiments are shown. **(e,f)** Stability of MVA-ZIKV. MVA-ZIKV (P2 stock) was continuously grown in DF-1 cells to passage 9 (**e**) and at passage 9, 24 individual plaques were picked (**f**). Virus stocks from each passage and from the 24 individual plaques were used to infect cells and the expression of ZIKV prM and E proteins was determined by Western blotting. Rabbit anti-VACV E3 protein antibody was used as a VACV loading control. Arrows on the right indicate the position of the ZIKV prM and E proteins, and the VACV E3 protein. The sizes of standards (in kDa) are indicated on the left.

To generate novel vaccines against ZIKV that could activate the ZIKV-specific B- and T-cell immune responses, we have generated an MVA-based vaccine candidate encoding for the ZIKV prM-E structural genes (termed MVA-ZIKV). ZIKV prM-E structural genes of the ZIKV isolate Z1106033 (Suriname; the most contemporary American isolate available at the time we initiated this work)^8^, were inserted into the vaccinia virus (VACV) thymidine kinase (TK) locus of an optimized parental MVA (termed MVA-Δ-GFP) containing deletions in the VACV immunomodulatory genes *C6L*, *K7R*, and *A46R*^9,10^, and their expression is under the transcriptional control of a novel optimized synthetic Late/Early (pLEO160) promoter^11,12^ (Fig. 1a) (see Methods).

The correct generation of MVA-ZIKV was analyzed by PCR using oligonucleotides annealing in the VACV TK-flanking regions that demonstrated the proper insertion of the ZIKV prM-E structural genes within the genome of MVA-ZIKV, with no wild-type MVA virus contamination (Fig. 1b). Moreover, the correct nucleotide sequence of the ZIKV prM-E genes inserted in the VACV TK locus was further confirmed by DNA sequencing (data not shown).

To demonstrate that MVA-ZIKV constitutively expresses and correctly processes the ZIKV prM-E polyprotein into prM and E structural proteins, we performed a Western blot analysis of cell extracts from MVA-infective permissive chicken DF-1 cells, mock infected or infected with MVA-ZIKV, parental MVA-Δ-GFP, or attenuated wild-type (WT) MVA (MVA-WT) using specific antibodies that recognize the ZIKV prM and E proteins. The results proved that MVA-ZIKV correctly expressed the ZIKV prM-E polyprotein that was properly processed leading to the ZIKV prM and E proteins of expected molecular sizes (Fig. 1c). The presence of two bands in the western blot using the anti-ZIKV E antibody is compatible with the production of different ZIKV E protein species that differ in post-translational modification^13^, being the upper band compatible with the full-length glycosylated E protein.

To analyze whether expression of ZIKV prM-E structural proteins affects MVA replication in cell culture, we evaluated the growth kinetics of MVA-ZIKV and MVA-WT in permissive DF-1 cells. Both viruses had a similar kinetics of viral growth (Fig. 1d), demonstrating that the constitutive expression of ZIKV prM-E structural proteins does not weaken MVA vector replication under permissive conditions. Moreover, similarly to parental MVA-WT and as expected, MVA-ZIKV is a viral vector that does not replicate in human HeLa cells (Fig. 1d).

Next, to ensure that MVA-ZIKV is stable and the insert can be maintained in the viral genome without the loss of the sequence encoding the ZIKV prM-E structural genes, MVA-ZIKV was grown in DF-1 cells infected at low multiplicity of infection (MOI) for 9 successive passages, and expression of the ZIKV prM and E proteins was determined by Western blotting (Fig. 1e). The experiment revealed that MVA-ZIKV efficiently expresses the ZIKV prM and E proteins after consecutive passages. Moreover, analysis by Western blot of the expression of the ZIKV E protein in 24 individual plaques isolated from MVA-ZIKV at passage 9 showed that 100% of the plaques correctly expressed the ZIKV E protein (Fig. 1f), demonstrating the high genetic stability of MVA-ZIKV.

**Figure 2:**
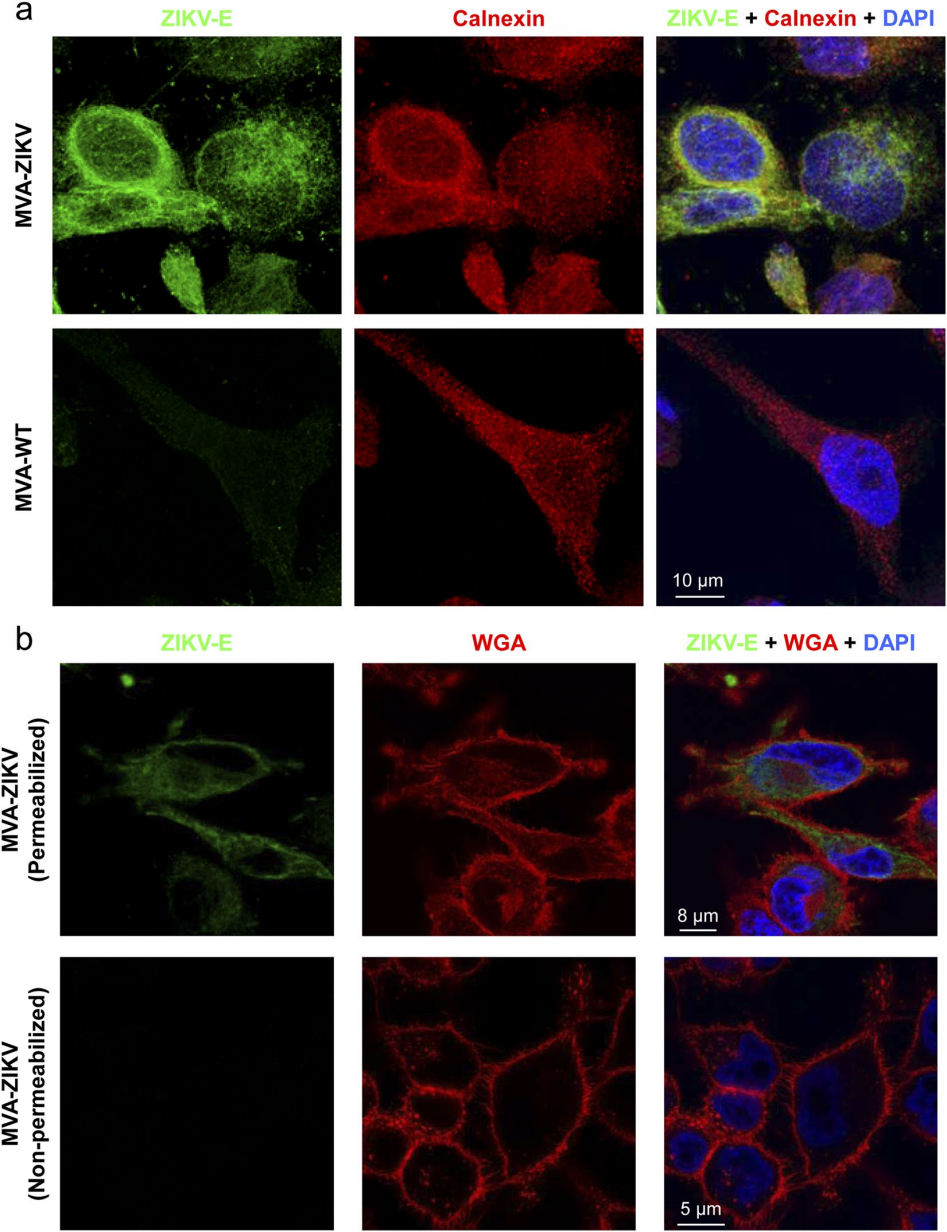
Immunofluorescence analysis of the expression of ZIKV E protein by MVA-ZIKV. **(a)** Detection of ZIKV E protein in the ER. HeLa cells were infected at 0.5 PFU/cell with MVA-ZIKV or MVA-WT for 24 h. Then, permeabilized cells were labeled with an anti-ZIKV E monoclonal mouse antibody and an anti-calnexin antibody. Anti-ZIKV E was detected with a mouse secondary antibody conjugated with the fluorochrome Alexa Fluor 488 (green). Anti-calnexin was detected with a rabbit secondary antibody conjugated with Alexa Fluor 594 (red). Cell nuclei were stained using DAPI (blue). The degree of co-localization of E and calnexin proteins is shown on the right by the yellow color. Scale bar: 10 μm. **(b)** Immunofluorescence analysis of ZIKV E protein in the cell membrane. HeLa cells were infected at 0.5 PFU/cell with MVA-ZIKV and at 24 h permebilized (upper panels) or non-permebilized (lower panels) fixed cells were stained with WGA probe conjugated to the fluorescent dye Alexa Fluor 594 (red) and a mouse monoclonal anti-ZIKV E antibody further detected with a mouse secondary antibody conjugated with the fluorochrome Alexa Fluor 488 (green). Cell nuclei were stained using DAPI (blue). Scale bars: 5 μm and 8 μm.

Flavivirus prM and E proteins are synthesized at the endoplasmic reticulum (ER)^14^. Thus, the expression and intracellular localization of the ZIKV E protein expressed by MVA-ZIKV was studied by confocal immunofluorescence microscopy in non-permissive HeLa cells infected with MVA-ZIKV and MVA-WT using an antibody against ZIKV E protein and a specific antibody to detect ER (anti-Calnexin) (Fig. 2a). The results showed that at 24 h post-infection ZIKV E protein (in green) was highly expressed from MVA-ZIKV-infected cells and, as expected, co-localized with the ER (Fig. 2a, right panel). Moreover, to determine whether ZIKV E protein could be detected on the cell surface, HeLa cells were infected with MVA-ZIKV and permeabilized and non-permeabilized cells were analyzed by confocal immunofluorescence microscopy using an antibody against ZIKV E protein and the wheat germ agglutinin (WGA) probe to label the surface of fixed cells (Fig. 2b). While the infected-cell membrane was well observed using the WGA probe, we did not detect labeling of ZIKV E protein. These results indicate that, as expected, the ZIKV E protein is not present on the surface of infected cells, and suggest that is release to the medium.

In summary, ZIKV prM and E structural proteins are expressed efficiently by MVA-ZIKV in cultured cells.

### MVA-ZIKV produced VLPs

**Figure 3:**
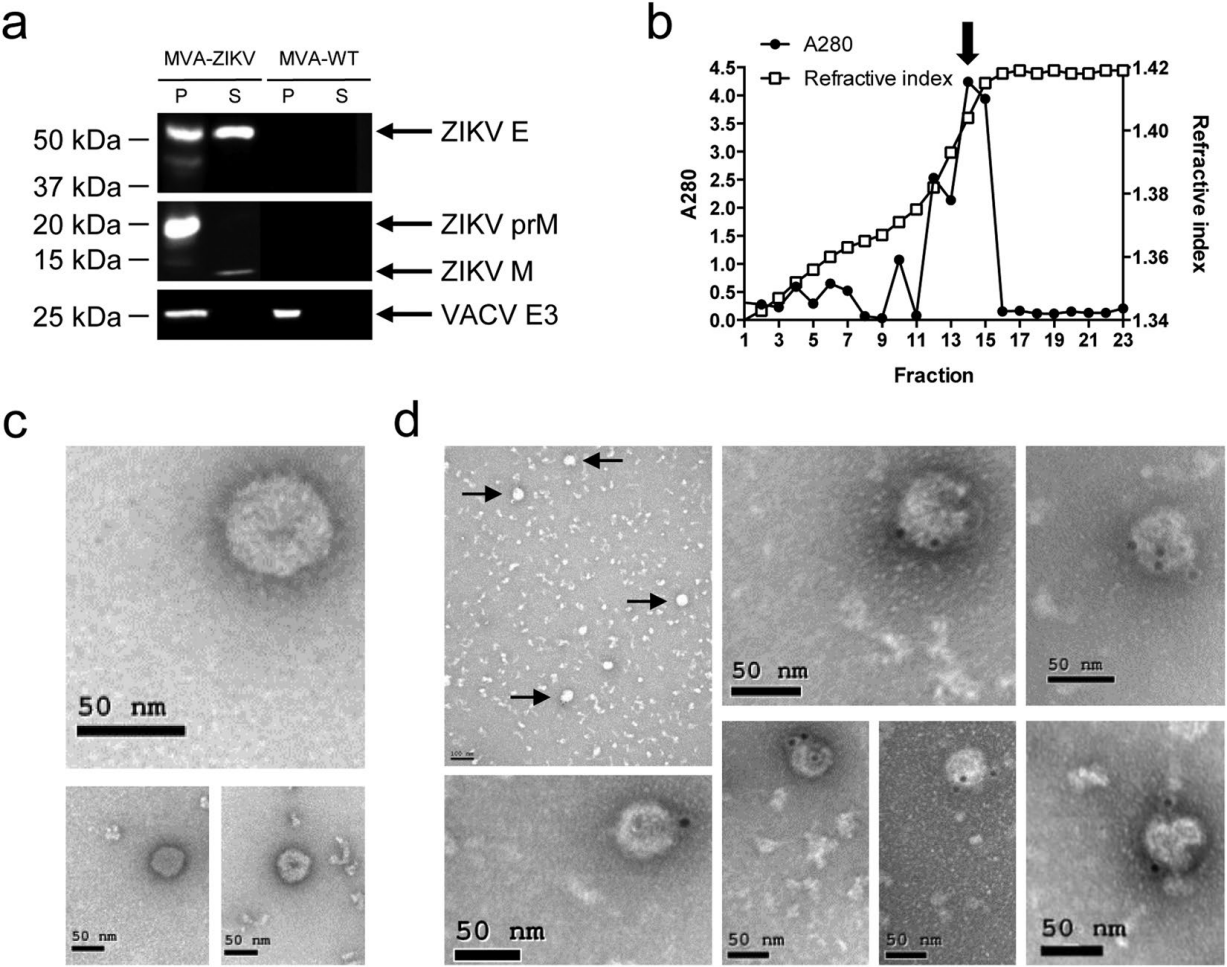
MVA-ZIKV produced VLPs. (**a**) Western blot analysis of the ZIKV proteins detected in cells extracts (P) or in supernatants (S) concentrated through a 20% sucrose cushion and derived from HeLa cells infected with MVA-ZIKV or MVA-WT. Arrows on the right indicate the positions of the ZIKV prM, M and E proteins. The sizes of standards (in kDa) are indicated on the left. **(b)** Amount of protein and sucrose density in fractions obtained after ultracentrifugation of MVA-ZIKV-concentrated supernatants loaded into a 20–60% w/v sucrose gradient. The amount of protein in each fraction was determined by spectrophotometry measuring the absorbance at 280 nm (A280). The sucrose density in each fraction was determined by refractometry. Arrow indicates the fraction (14) analyzed by electron microscopy. **(c,d)** Detection by electron microscopy of VLPs produced by MVA-ZIKV. Negative-stained (**c**) or anti-ZIKV E immunogold-stained (**d**) transmission electron microscopy images of purified ZIKV VLPs contained in the fraction 14 of the MVA-ZIKV gradient shown in (**b**). Arrows indicate the VLPs detected in a lower magnification image of the anti-ZIKV E immunogold-stained assay. Scale bars: 50 nm or 100 nm.

It has been described that coexpression of flaviviral prM and E proteins results in the production of VLPs [also termed subviral particles (SVPs)]^13,15–18^. Thus, to test whether MVA-ZIKV could form VLPs, we investigated their presence in the supernatant of infected cells. Therefore, HeLa cells were infected with MVA-ZIKV and MVA-WT and at 18 h post-infection, supernatants were concentrated by pelleting through a 20% sucrose cushion. The analysis by western blot of the concentrated supernatants demonstrated the presence of ZIKV M and E proteins, confirming their release to the medium (Fig. 3a). Moreover, the detection of the mature M protein suggests that fully-assembled VLPs could be produced (Fig. 3a). Thus, next we loaded the concentrated supernatants into a 20–60% w/v sucrose gradient and after ultracentrifugation, fractions were taken and the amount of protein and sucrose density was analyzed (Fig. 3b). A single peak of protein, determined by measurement of the absorbance of each fraction at 280 nm, exhibited a density about 1.18 g/cm^3^ (42% of sucrose) estimated by refractometry analysis. This value is comparable to that exhibited by other VLPs from different flaviviruses^19–22^. The protein peak fraction was then analyzed by negative staining and transmission electron microscopy to evaluate whether VLPs were observed (Fig. 3c). The results showed that MVA-ZIKV formed smooth spherical particles of similar size (around 50 nm) and morphology than ZIKV VLPs produced by other ZIKV vaccines expressing prM-E genes^13,15,18,23^ (Fig. 3c). Immunogold electron microscopy demonstrated the presence of ZIKV E protein on the surface of these structures, confirming that these particles were ZIKV VLPs (Fig. 3d).

In summary, MVA-ZIKV expresses ZIKV prM and E proteins that can be assembled into VLPs secreted from the infected cells.

### MVA-ZIKV is highly immunogenic in immunocompetent mice

**Figure 4:**
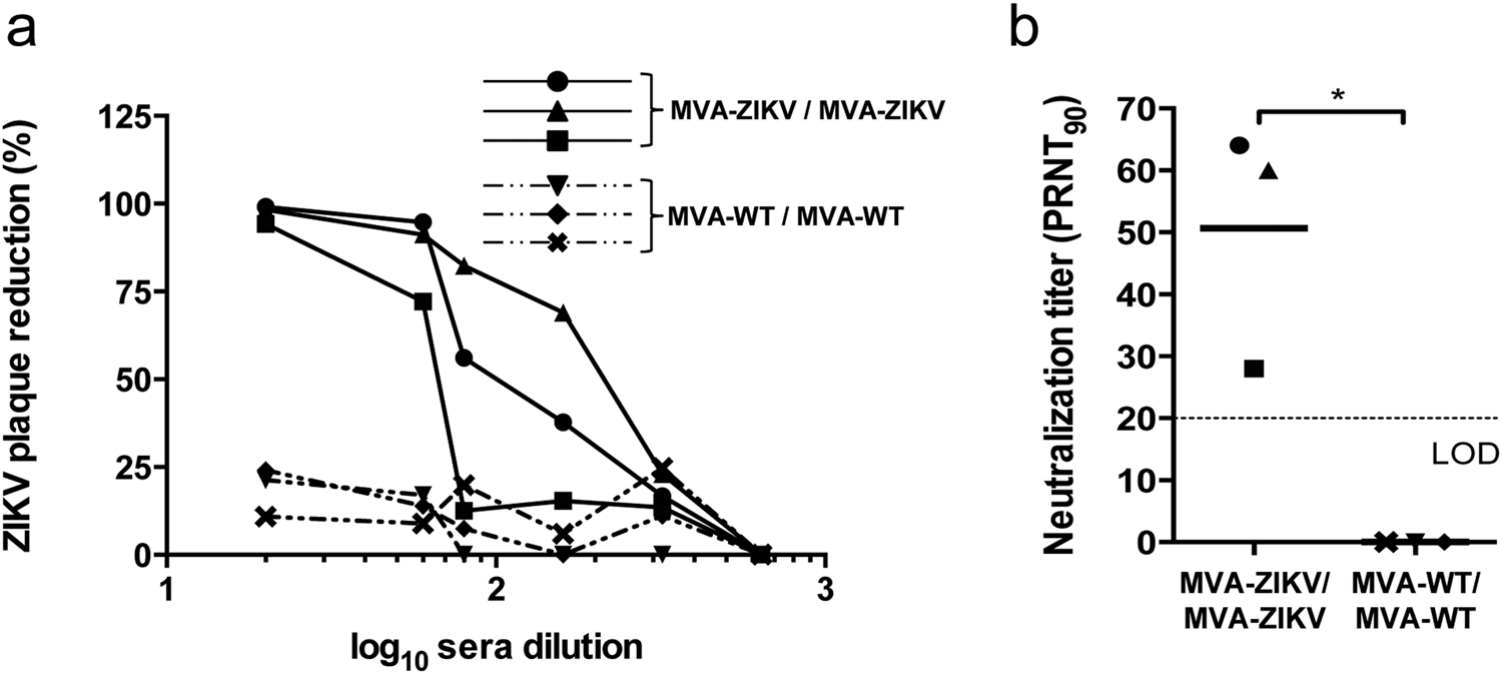
Induction of neutralizing antibodies by MVA-ZIKV in immunocompetent mice. Balb/c mice were immunized with MVA-ZIKV/MVA-ZIKV or MVA-WT/MVA-WT and 10 days after the last immunization ZIKV-specific humoral immune responses were analyzed (see Methods). **(a)** Percentage of ZIKV plaque reduction. Data represent the percentage of ZIKV plaque reduction, determined by a PRNT assay, from each individual serum sample at different serial dilutions. **(b)** ZIKV-neutralizing antibody titers. Data represent the reciprocal of the serum dilution that inhibited plaque formation by 90% (PRNT_90_), relative to samples incubated with negative control sera. Dashed line indicates the limit of detection (LOD) of the neutralization assay (1/20 dilution). The statistically significant difference between both groups is indicated (*P < 0.05).

Neutralizing antibodies against ZIKV are critical to control ZIKV infection^24–26^. Thus, to evaluate whether MVA-ZIKV is able to induce neutralizing antibodies against ZIKV, we determined the plaque reduction neutralization titers (PRNT) against ZIKV in serum samples obtained at 10 days post-boost from Balb/c mice immunized with MVA-ZIKV or MVA-WT (negative control group), following a homologous prime/boost immunization protocol (see Methods). The results showed that individual serum obtained from mice immunized with MVA-ZIKV neutralized ZIKV (PA259459 strain, from Panama) in a dilution-dependent manner (Fig. 4a), compared to serum from MVA-WT-immunized animals where no neutralization was observed. The elicited mean-value antibody titers induced by MVA-ZIKV neutralized 90% of ZIKV (PRNT_90_) at a dilution of 1/50 (Fig. 4b).

**Figure 5:**
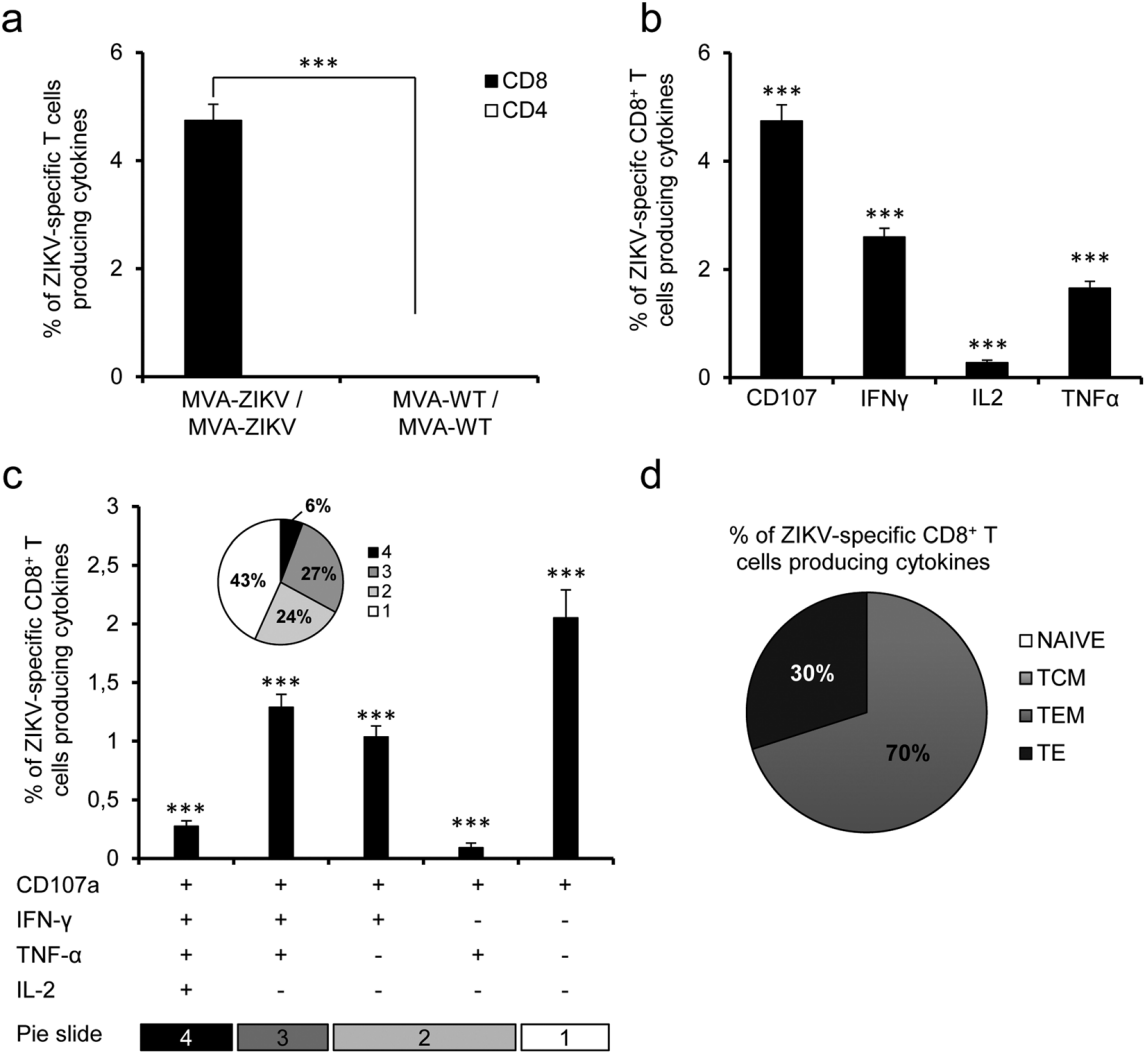
MVA-ZIKV induced potent CD8^+^ T cell responses in mice. Balb/c mice were immunized as in Fig. 4. P values indicate significantly higher responses in comparison of MVA-ZIKV/MVA-ZIKV to MVA-WT/MVA-WT (***P < 0.001). **(a)** Overall magnitude of ZIKV-specific CD4^+^ and CD8^+^ T cells. The values represent the sums of the percentages of T cells producing CD107a and/or IFN-γ and/or TNF-α and/or IL-2 against the ZIKV E protein peptide pool. **(b)** Pattern of ZIKV-specific CD8^+^ T cell immune responses in MVA-ZIKV-vaccinated mice. Frequencies were calculated by reporting the number of CD8^+^ T cells producing CD107a or IFN-γ or TNF-α or IL-2. **(c)** Polyfunctional profile of ZIKV-specific CD8^+^ T cell immune responses in MVA-ZIKV-vaccinated mice. Those T cell populations with a positive response are shown on the x axis, while the percentages of CD8^+^ T cells producing CD107a and/or IFN-γ and/or TNF-α and/or IL-2 against the ZIKV E peptide pool are shown on the y axis. Responses are grouped and coded on the basis of the number of functions (4, 3, 2, or 1). The pie charts summarize the data, with each slice corresponding to the proportion of ZIKV-specific CD8^+^ T cells exhibiting one, two, three, or four functions within the total population of ZIKV-specific CD8^+^ T cells. **(d)** Phenotypic profile of ZIKV-specific CD8^+^ T cells in MVA-ZIKV-vaccinated mice. CD127 and CD62L expression was used to identify naive, TCM, TEM and TE subpopulations. Each slice corresponds to the proportion of each ZIKV-specific CD8^+^ T cell subpopulations within the total ZIKV-specific CD8^+^ T cells producing CD107a and/or IFN-γ and/or TNF-α and/or IL-2.

Although the specificity of CD8^+^ T cell responses varies among ZIKV strains, CD8^+^ T cells might play a protective role against ZIKV infection^27–30^. Therefore, to investigate in detail the capability of MVA-ZIKV to stimulate T cellular immune responses against ZIKV, we next evaluated the ZIKV-specific T cell immune responses elicited by MVA-ZIKV in Balb/c mice immunized following a homologous prime/boost immunization protocol. ZIKV E-specific CD4^+^ and CD8^+^ T cell immune responses induced by MVA-WT/MVA-WT and MVA-ZIKV/MVA-ZIKV immunization groups were measured at 10 days post-boost by an intracellular cytokine staining (ICS) assay, after the stimulation of splenocytes with ZIKV-specific peptide pools spanning the entire ZIKV E protein (Fig. 5). The results showed that immunization with MVA-ZIKV stimulated robust ZIKV-E-specific CD8^+^ T cell immune responses (determined as the percentage of ZIKV E-specific CD8^+^ T cells producing IFN-γ, TNF-α, and/or IL-2 cytokines, as well as the expression of CD107a on the surface of activated T cells as an indirect marker of cytotoxicity) (Fig. 5a) (ZIKV-specific CD8^+^ T cell responses in estimated absolute numbers are included in Table S1). MVA-ZIKV elicited total ZIKV-specific immune responses mediated mostly by CD8^+^ T cells, with very low levels of ZIKV-specific CD4^+^ T cells (Fig. 5a). ZIKV-specific CD8^+^ T cells produced mainly CD107a, followed by similar levels of IFN-γ and TNF-α, and to a minor extent IL-2 (Fig. 5b). The quality of the ZIKV-specific T cell immune response was defined by the pattern of cytokine production (IFN-γ, TNF-α, and/or IL-2) and its cytotoxic potential (CD107a). Thus, the most representative ZIKV-specific CD8^+^ T cell populations induced by MVA-ZIKV were those producing CD107a + IFN-γ + TNF-α (triple), CD107a + IFN-γ (double) and CD107a (single), with a high polyfunctional pattern represented by 57% of CD8^+^ T cells having two, three, or four functions (Fig. 5c).

Moreover, we also determined the phenotype of the ZIKV-specific CD8^+^ T cells by evaluating the presence of CD127 and CD62L surface markers, which define memory subpopulations: T central memory (TCM; CD127^+^/CD62L^+^), T effector memory (TEM; CD127^+^/CD62L^−^), and T effector (TE; CD127^−^/CD62L^−^) T cells^31^. The results showed that immunization with MVA-ZIKV induced ZIKV-specific CD8^+^ T cells with a phenotype of TEM (70%) and TE (30%) (Fig. 5d).

In summary, MVA-ZIKV is highly immunogenic in immunized mice inducing neutralizing antibodies against ZIKV and potent and polyfunctional ZIKV-specific CD8^+^ T cell immune responses mainly with TEM and TE phenotypes.

### MVA-ZIKV controls viral replication in a challenged mouse model

**Figure 6:**
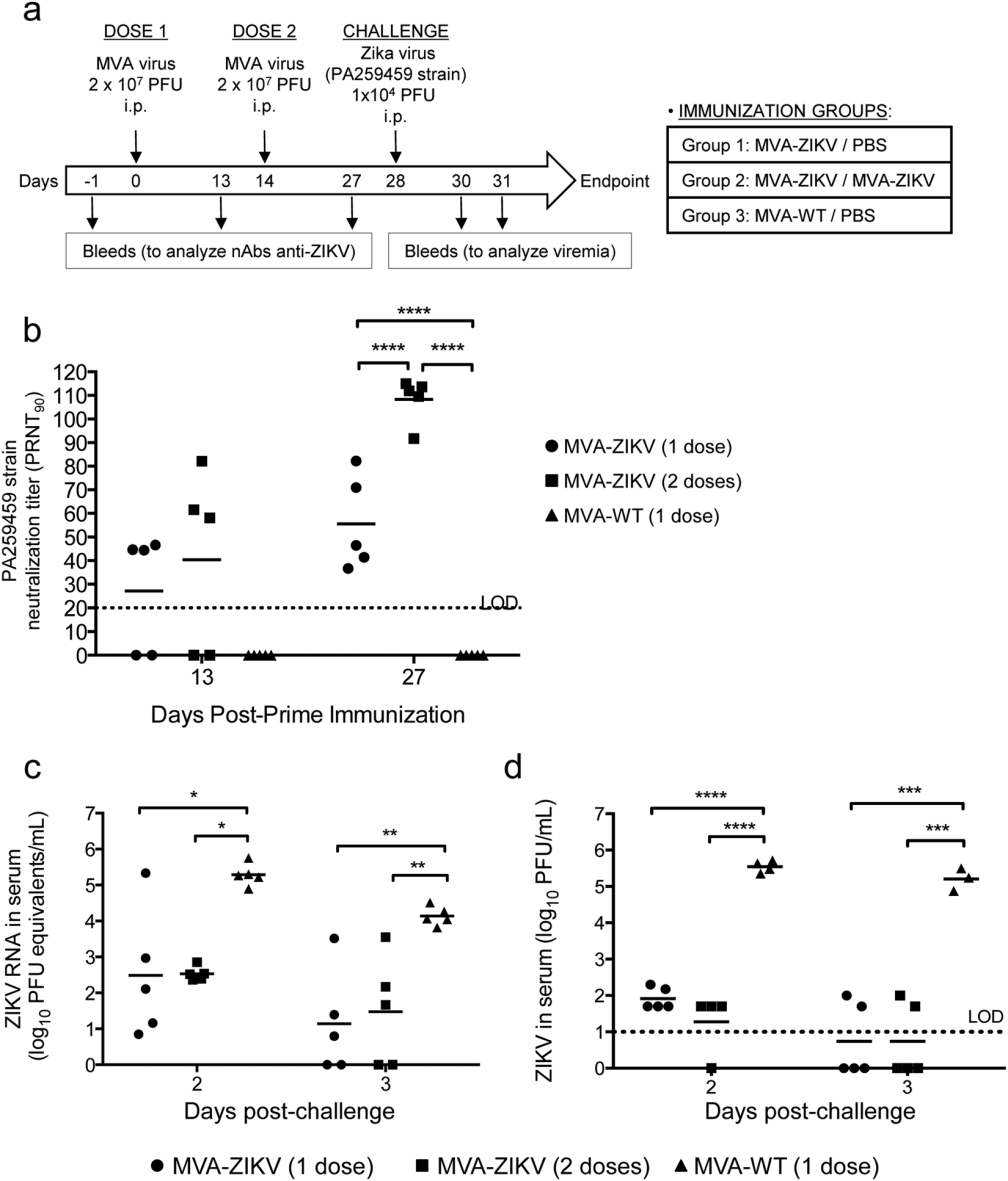
Efficacy of MVA-ZIKV in immunocompromised susceptible IFNAR^−/−^ mice. (**a**) Inoculation scheme. Groups of IFNAR^−/−^ mice (n = 10 mice/group) were immunized with 2 × 10^7^ PFUs of MVA-WT (one dose, at day 0) or MVA-ZIKV (one or two doses, at days 0 and 14, respectively) by the i.p. route. Twenty eight days after the first immunization, mice were challenged with 10^4^ PFUs of ZIKV (PA259459, strain Panama) via the i.p. route (see Methods). **(b)** ZIKV-neutralizing antibody titers (PRNT_90_) detected at 13 and 27 days post-prime immunization in sera (n = 5) of animals immunized with one dose of MVA-WT or one or two doses of MVA-ZIKV. Titers of neutralizing antibodies against ZIKV PA259459 strain were determined by a PRNT assay and are expressed as the reciprocal of the serum dilution that inhibited plaque formation by 90% (PRNT_90_), relative to samples incubated with negative control sera (from day 1 pre-prime). Dashed line indicates the limit of detection (LOD) of the neutralization assay (1/20 dilution). The statistically significant difference between the groups is indicated (****P < 0.0001). **(c)** ZIKV RNA viremia after challenge. Blood samples were collected at days 2 (n = 5) or 3 (n = 5) post-challenge, and ZIKV RNA viremia was analyzed by quantitative real-time PCR (PFU equivalents/ml). Graph shows mean with each point representing an individual mouse. P values indicate significantly higher responses between the different groups (*P < 0.05; **P < 0.005). **(d)** ZIKV infectious virus after challenge. Blood samples were collected at days 2 (n = 5) or 3 (n = 5) post-challenge, and ZIKV infectious virus was analyzed by a plaque assay (PFUs/ml). Graph shows mean with each point representing an individual mouse. P values indicate significantly higher responses between the different groups (***P < 0.001, ****P < 0.0001).

The efficacy of MVA-ZIKV as a vaccine candidate against ZIKV was studied in mice deficient in the α/β interferon receptor (IFNAR^−/−^), a suitable susceptible mouse model for ZIKV^32,33^. Thus, six-week-old IFNAR^−/−^ mice were immunized by intraperitoneal (i.p.) route with MVA-ZIKV (one or two doses) or MVA-WT (one dose; used as a control), at days 0 and/or 14. At day 28 mice were challenged with 10^4^ PFUs of ZIKV (PA259459 strain, from Panama) (Fig. 6a). Blood was obtained at days 13 and 27 (before challenge) to analyze the neutralizing antibodies in sera, and 2 and 3 days after virus challenge to analyze viremia. The results showed that one or two doses of MVA-ZIKV elicited good neutralizing antibody titers against ZIKV PA259459 strain, with a second dose significantly increasing the ZIKV neutralization titer (PRNT_90_) to around 110 (Fig. 6b). Furthermore, neutralization of another ZIKV strain from the Asian-American lineage (FSS13025, Cambodia 2010) using serum samples obtained after two doses of the MVA-ZIKV vaccine showed similar results (PRNT_90_ 149 ± 77), confirming that the elicited neutralizing antibodies could also neutralize other ZIKV strains. Next, the MVA-ZIKV efficacy was analyzed in immunized mice after challenge with ZIKV. Intraperitoneal infection of adult IFNAR^−/−^ mice (10-week-old) with the ZIKV American strain PA259459 did not result in adverse body weight loss or mortality, in spite of induced viremia (Fig. 6c,d). Along this line, the MVA-ZIKV efficacy, defined as the capacity to control ZIKV replication, was evaluated at days 2 and 3 post-challenge determining in serum ZIKV viremia by quantitative RT-PCR (Fig. 6c) and presence of ZIKV infectious virus by plaque assay (Fig. 6d). The results showed that MVA-ZIKV-vaccinated mice control ZIKV replication as index of significant reduction in levels of ZIKV RNA (Fig. 6c) and of infectious virus (Fig. 6d), compared to the control group MVA-WT. One or two doses of MVA-ZIKV reduced the ZIKV viremia and infectious virus by 2.5–4 logs, while MVA-WT-immunized mice developed ZIKV infection, with high levels of ZIKV RNA and of infectious virus that peaked at day 2 post-challenge (Fig. 6c,d).

In conclusion, MVA-ZIKV is effective in controlling ZIKV replication in a susceptible mouse model for ZIKV infection, even with a single vaccine dose.

## Discussion

ZIKV is an important emerging flavivirus transmitted by infected mosquitoes from the genus *Aedes* that can cause severe complications in humans. The virus has caused recent outbreaks of the disease worldwide and their future expansion to novel geographical areas is highly possible^2,3^. Although several ZIKV vaccine candidates have been generated using different strategies^4,5^, actually no licensed vaccine exists. These vaccines have been tested in animals in preclinical trials and in early phase I or II human clinical trials, but although they are immunogenic many of them required numerous immunizations, have high costs of manufacturing or require the use of chemical adjuvants. Therefore, novel strategies are necessary to achieve potent and long-lasting protective immune responses, with less immunizations, better safety profile, and lower costs of vaccine production. An ideal ZIKV vaccine should (1) be safe, especially for women of child-bearing age; (2) be stable and cost effective to manufacture, as ZIKV is endemic in developing countries; (3) avoid the addition of chemical adjuvants to minimize associated-risks; (4) be effective against all circulating ZIKV strains; and (5) be able to induce a rapid onset of protective levels of neutralizing anti-ZIKV antibodies as well as T cell responses to control the replication of the virus.

In this report, we have developed a novel, immunogenic and effective ZIKV vaccine candidate (termed MVA-ZIKV) based on the highly attenuated poxvirus vector MVA expressing the ZIKV prM and E structural proteins that are assemble into VLPs. MVA-ZIKV induced potent ZIKV-specific B and T cell immune responses in immunized mice and controlled ZIKV replication in a susceptible immunocompromised mouse model for ZIKV infection challenged with ZIKV. This is the first report of an MVA-based vaccine against ZIKV expressing the prM-E structural proteins (the main immunogenic antigens of ZIKV) and forming VLPs. MVA-ZIKV possesses many of the characteristics needed for an ideal vaccine against ZIKV. MVA-ZIKV is replication competent in avian cells but replication deficient in human cells, making it safe for humans. The MVA safety profile has been demonstrated by its use as a variola vaccine during years and by extensive clinical trials testing MVA recombinants as vaccine vector against different infectious diseases^6,7,34^, confirming that MVA vaccines are safe even for immunocompromised individuals, children and elderly. Moreover, a recent study in pregnant macaques immunized with an MVA vector showed no adverse or teratogenic effects^35^, opening the possibility of its use in pregnant women. This observation could be highly relevant in ZIKV vaccination because of severe sequelae of ZIKV infection during pregnancy. Additionally, it has been reported that VACV induced strong innate responses during vaccination acting itself as an adjuvant^36^, eliminating the need of other chemical adjuvants, minimizing reactogenicity and adverse effects caused by these synthetic substances^37,38^, and promoting a better activation of the immune system^39^. Furthermore, MVA vaccines can be produced easily in primary avian cells, the most common cells used in the GMP manufactured of MVA lots, and the cost of production and manufacture is low. Therefore, the use of an MVA vector expressing ZIKV antigens, such as the MVA-ZIKV reported here, is a promising approach against ZIKV. In addition, the MVA-ZIKV vaccine candidate that we have developed has a MVA genome more optimized than its parental MVA-WT, due to additional deletions in 3 MVA immunomodulatory genes (*C6L*, *K7R* and *A46R*), whose deletion enhanced in immunized mice the HIV-1-specific cellular and humoral immune responses induced by a MVA vector expressing Env, Gag, Pol and Nef HIV-1 antigens^40^. Furthermore, one dose of a vaccine against chikungunya virus (CHIKV) based on MVA with these 3 deletions and expressing CHIKV structural antigens (termed MVA-CHIKV) protected 100% of animals against CHIKV infection in mice and nonhuman primates^9,41^. Recently, using this optimized MVA vector we described MVA-based vaccines that protected against Ebolavirus (EBOV) infection in a susceptible mouse model^10^, reinforcing the use of this vaccine platform against emerging viruses, like CHIKV, EBOV and ZIKV.

Our MVA-ZIKV vaccine candidate expresses the ZIKV prM and E structural genes of the ZIKV isolate Z1106033 (derived from an Asian-American lineage virus, isolated from a patient in Suriname). Although there are 3 main ZIKV lineages (East African, West African, and Asian-American)^42^, up to date, only one ZIKV serotype has been established^17^. Thus, MVA-ZIKV vaccine should be potentially effective against different ZIKV strains, due to low heterogeneity between ZIKV lineages. In fact, our results demonstrate that elicited neutralizing antibodies from MVA-ZIKV are effective against a different ZIKV strain of the Asian-American lineage. The E protein is the main ZIKV protein involved in receptor binding and fusion, while the M protein is a small protein that is hidden under the E protein layer^43,44^. The prM and E proteins are, together with the inactivated whole viruses, the flaviviral proteins more widely used as vaccine antigens showing an excellent capacity to protect against this family of virus, including ZIKV^4,5^. Recent studies comparing in mice and nonhuman primates the efficacy of diverse vectored-based vaccines against ZIKV, showed that live-attenuated adenoviral vectors are one of the most potent approaches, even in long-term studies^24,45,46^. These results confirm the advantage of live-attenuated recombinant vaccines, a case that can be extrapolated to the MVA-ZIKV vaccine developed here.

Another advantage of the MVA-ZIKV vaccine candidate is that it expresses high levels of ZIKV prM and E antigens, as observed by Western blot and/or immunofluorescence. This high expression is due to the presence in the MVA vector of an optimized strong VACV late/early promoter previously described^11,12^. The ZIKV E protein expressed by MVA-ZIKV co-localized with the ER (the place where prM-E protein is translocated to dimerize), supporting their correct subcellular localization. Furthermore, the apparent absence of the ZIKV E protein in the cell membrane of the MVA-ZIKV-infected cells suggests that the E protein is not exposed in the outer membrane, but released to the medium. Moreover, we have detected the presence of the ZIKV E protein and the mature M protein in supernatants of cells infected with MVA-ZIKV, confirming their release to the medium. Remarkably, the analysis by immunogold electron microscopy of the purified supernatants demonstrated that MVA-ZIKV produced VLPs, similarly to other ZIKV vaccines or flavivirus expressing the prM-E genes^13,15,18,23^. Furthermore, an additional advantage as a vector is that MVA-ZIKV is highly stable in cell culture, maintaining the expression of ZIKV antigens at least during 9 continuous passages at low MOI, with 100% of the plaques picked at passage 9 expressing the correct size ZIKV antigens.

The adaptive immune response against ZIKV plays an essential role in regulating ZIKV infection and preventing the spread of the virus to key organs, like the brain and testes^27,46^, especially when type I IFN response is reduced, as in human ZIKV infection^47^. It has been described that neutralizing antibody titers higher than 10 correlated with protection after vaccination with most of the licensed flavivirus vaccines^48^, and adoptive transfer studies suggest that vaccine-elicited antibodies, even at low titers, are sufficient for protection against ZIKV challenge in mice and nonhuman primates^24–26^. Our MVA-ZIKV, after 2 doses and at 10–13 days post-boost, induced antibody titers (PRNT_90_) of 50 or higher than 100 in WT or IFNAR^−/−^ mice, respectively, in the range of a good and immunogenic vaccine against ZIKV.

On the other hand, to date relatively little is known regarding the T cell response to ZIKV infection. It has been recently shown that adoptive transfer of ZIKV-specific CD8^+^ T cells prevented disease in immunocompromised susceptible mice^27,29^. Moreover, given the neurotropism of ZIKV and its presence in fetal brain tissues and cerebrospinal fluid in both humans and animal models^49^, CD8^+^ T cells may have a key role in clearing ZIKV from the central nervous system (CNS) and, thus, in preventing or mitigating neurological complications. A recent study in pregnant macaques infected with ZIKV and then treated with a cocktail of ZIKV-neutralizing human monoclonal antibodies (mAbs) at the peak of viremia, showed that while the mAbs can be effective in clearing the virus from the maternal sera of treated nonhuman primates, it is not sufficient to fully stop vertical transmission^50^. This finding suggest the importance of CD8^+^ T cell immune response against ZIKV infection, especially when the immune system integrity is compromised, as during pregnancy^51^. These results suggest that a vaccine eliciting ZIKV-specific CD8^+^ T cell responses could contribute effectively at preventing disease and clearing the virus from the CNS. Up to now only few vaccine candidates have described the induction of ZIKV-specific T cell responses towards E protein; reporting mainly a modest activation^24,25,46,52^. In our case and using immunocompetent mice, MVA-ZIKV is able to induce potent and polyfunctional ZIKV-specific CD8^+^ T cell immune responses, as natural ZIKV infection does^29^, indicating that MVA-ZIKV could be a promising vaccine candidate against ZIKV. Furthermore, the low CD4^+^ T cell response that we observed was consistent with a report where appearance of CD4^+^ T cell responses in ZIKV-infected rhesus monkeys was not found until production of antibodies and CD8^+^ T cell responses, at 2 weeks post-infection^53^.

Therefore, all these results highlight the need for the development of vaccines that drive a potent T cell and humoral antibody responses, as MVA-ZIKV, in order to protect against ZIKV infection and spread.

Current immunocompetent mouse models for ZIKV are not susceptible to viremia or lethal outcome. Hence, to analyze the efficacy of MVA-ZIKV we used a type I IFN receptor-knockout mouse model (IFNAR^−/−^) that is extensively used as a challenge model for ZIKV vaccine candidates^32,33^. As it happened in immunocompetent Balb/c mice, IFNAR^−/−^ mice immunized with one or two doses of MVA-ZIKV produced ZIKV-neutralizing antibodies (against ZIKV PA259459 strain or against other isolates such as FSS13025) and controlled ZIKV viral replication after a challenge with live ZIKV PA259459 strain Panama, representative of the circulating virus during the American epidemic. The potent generation of neutralizing antibodies by MVA-ZIKV could be due to the production of VLPs, which are efficiently recognized by the B cells^54^, leading to an MHC-II upregulation that will favor the production of high levels of neutralizing antibodies against ZIKV. VLPs are highly immunogenic and therefore are very important to improve the ZIKV-specific immune responses *in vivo*, as it has been reported with VLPs from ZIKV or other viruses that induced high titers of neutralizing antibodies which correlate with protection^13,15,18,23,55^. From a vaccine point of view, we suggest that in order to further enhance *in vivo* the production of ZIKV neutralizing antibodies, it might be convenient to perform immunizations with the combined MVA-ZIKV and purified VLPs as immunogens.

We observed that after ZIKV challenge infection immunized IFNAR^−/−^ mice (10-week-old at the moment of challenge) did not loss body weight or have mortality during 15 days post-challenge. Our results are consistent with data obtained in adult IFNAR^−/−^ mice (10–11-week-old) challenged with other ZIKV strains belonging to the same genetic lineage than PA259459 strain that did not develop disease and had higher survival rates than young mice challenged with the same ZIKV strain^32,33,56,57^. Accordingly, we selected this non-lethal challenge model for the evaluation of vaccine efficacy because it resembles better the ZIKV infection in humans, which usually provokes a mild disease that is not fatal.

The control of ZIKV infection obtained after administration of one or two doses of MVA-ZIKV was in the range between 2.5 and 4 log virus reduction, highlighting the efficacy of the vaccine. We do not know the contribution of B and T cell responses in the control of ZIKV infection in this mouse model. However, considering the potent ZIKV-specific CD8^+^ T cellular immunogenicity and humoral immune responses induced by MVA-ZIKV in the immunocompetent mouse model, we suggest that in a similar way neutralizing antibodies and CD8^+^ T cells should both contribute to the effective reduction of viral load observed in IFNAR^−/−^ mice and this vaccine would be an effective approach to protect against ZIKV. Further studies will be needed to define in this system the independent role of B and T cell responses elicited by MVA-ZIKV in virus protection. Remarkably, the fact that MVA-ZIKV induced antibodies that can neutralize other ZIKV strain (FSS13025, Cambodia 2010), the high homology among ZIKV structural proteins from different isolates and the existence of only one ZIKV serotype^17^ support that the protection we observed could be likely extended to other different ZIKV strains. The protection of mice vaccinated with MVA-ZIKV against other distant ZIKV strains will be tested in future studies.

In summary, we have developed a novel and promising ZIKV vaccine candidate (MVA-ZIKV) that produced VLPs and in mice induced ZIKV-specific neutralizing antibodies and potent CD8^+^ T cell immune responses, being strongly effective in reducing ZIKV viremia after a challenge with ZIKV.

## Methods

### Ethics statement

The immunogenicity animal studies were approved by the Ethical Committee of Animal Experimentation (CEEA) of Centro Nacional de Biotecnología (CNB, Madrid, Spain) and by the Division of Animal Protection of the Comunidad de Madrid (PROEX 331/14) and were conducted at the CNB. The efficacy animal studies were approved by the CEEA of Instituto Nacional de Investigación y Tecnología Agraria y Alimentaria (INIA, Madrid, Spain) and by the Division of Animal Protection of the Comunidad de Madrid (PROEX 187/17) and were conducted in the biosafety level 3 laboratory at Centro de Investigación en Sanidad Animal (CISA-INIA, Madrid, Spain). Animal procedures were conformed to international guidelines and to the Spanish law under the Royal Decree (RD 53/2013). Animals were maintained and handled according to the recommendations of the CNB-CSIC and CISA-INIA institutional Ethics Committees.

### Cells

HeLa cells (a human epithelial cervix adenocarcinoma; ATCCCCL-2), DF-1 cells (a spontaneously immortalized chicken embryo fibroblast (CEF) cell line; ATCCCRL-12203) and primary CEF cells (obtained from specific-pathogen-free 11-day-old eggs; MSD, Salamanca, Spain) were grown in Dulbecco’s modified Eagle’s medium (DMEM) and 10% heat-inactivated fetal calf serum (FCS) (Gibco-Life Technologies), as previously described^58^. Vero cells (a kidney epithelial cell line from African green monkey; ATCCCCL-81) were grown in Eagle’s minimal essential medium (EMEM) and 5% heat-inactivated fetal bovine serum (Linus), as previously described^59^. Cell cultures were kept at 37 °C and 5% CO_2_ in a humidified incubator.

### Viruses

The parental VACV used for the generation of the recombinant MVA-ZIKV is an attenuated MVA-WT that was modified by inserting the green fluorescent protein (GFP) gene into the VACV TK locus and by deleting the immunomodulatory VACV genes *C6L*, *K7R*, and *A46R* (parental virus termed MVA-Δ-GFP)^9,10^. Then, the GFP gene present in the TK locus of MVA-Δ-GFP was replaced by the ZIKV prM-E genes to generate the MVA-ZIKV vaccine candidate (see later). We also include as control the MVA-WT. All MVAs were grown in primary CEF cells, purified, and titrated at least two times, as previously described^60^. All MVAs were free of contamination with mycoplasma, bacteria or fungi. ZIKV PA259459 and FSS13025 strains were isolated from an infected human in Panama in 2015 and in Cambodia in 2010, respectively. Both ZIKV isolates were propagated and titrated in semisolid agarose medium using Vero cells, as previously described^61^.

### ZIKV antigens

The ZIKV antigens inserted in the MVA-ZIKV vaccine candidate are the prM and E structural genes of ZIKV isolate Z1106033 (derived from an Asian-American lineage virus, isolated from a patient in Suriname at the onset of the late-2015 expansion of the virus in the Americas; GenBank accession number: KU312312)^8^. The prM and E are preceded by the last 18 amino acids of the C-terminal hydrophobic stretch of C-protein that acts as a signal peptide for the proper translocation of the prM protein into the lumen of the ER. The ZIKV prM-E sequence was chemically synthesized and codon optimized for human cell expression by GeneArt® (Thermo Fisher Scientific).

### Construction of pLEOLZ-ZIKV plasmid

The synthesized ZIKV prM-E genes were subcloned into the plasmid transfer vector pLEOLZ (Centro Nacional de Biotecnología) using the Gibson Assembly® technology (New England Biolabs), to obtain the pLEOLZ-ZIKV plasmid. The plasmid was amplified in ElectroMAX™ DH10B™ Cells (Thermo Fisher Scientific) and purified using a QIAGEN Plasmid Maxi Kit (Qiagen). Its correct generation was confirmed by DNA sequencing. Plasmid transfer vector pLEOLZ-ZIKV was used for the generation of recombinant MVA-ZIKV and allows the insertion of the ZIKV prM-E antigens in the TK locus of parental MVA-Δ-GFP by homologous recombination. It contains a novel synthetic VACV promoter termed pLEO160^11,12^, a multiple-cloning site, the human codon-optimized prM-E ZIKV genes introduced between the VACV TK-L and TK-R flanking regions, and the selectable marker genes for ampicillin and β-galactosidase (LacZ gene). The LacZ gene is inserted among two repetitions of the left TK-flanking region, allowing the deletion of the reporter LacZ gene from the final recombinant virus by homologous recombination after consecutive plaque purification steps.

### Generation of recombinant MVA-ZIKV

MVA-ZIKV was generated using MVA-Δ-GFP as parental virus and pLEOLZ-ZIKV as a plasmid transfer vector, using an infection/transfection protocol^9,10^. The recombinant virus obtained (termed MVA-ZIKV) was then grown in CEF cells, purified and titrated by plaque immunostaining assay^60^.

### PCR analysis of MVA-ZIKV

The correct generation and purity of recombinant MVA-ZIKV was confirmed by PCR using oligonucleotides TK-L and TK-R, annealing in the VACV left and right TK-flanking regions, respectively, and allowing the amplification of the ZIKV insert, as previously described^58,62,63^. Furthermore, the correct ZIKV insert was also confirmed by DNA sequencing.

### Analysis of MVA-ZIKV virus growth

To study the virus growth profile of MVA-ZIKV in comparison to that of MVA-WT, monolayers of permissive DF-1 cells or non-permissive HeLa cells were infected at 0.01 PFU/cell with MVA-WT or MVA-ZIKV, as previously described^9,10^. At different hours post-infection (hpi) (0, 24, 48, and 72) cells were harvested and virus titers in cell lysates were determined by a plaque immunostaining assay in DF-1 cells, as previously described^60^.

### Expression of ZIKV proteins from MVA-ZIKV by Western blot

To test the expression of the ZIKV antigens present in MVA-ZIKV, monolayers of DF-1 cells were mock infected or infected at 5 PFU/cell with MVA-WT, MVA-Δ-GFP, or MVA-ZIKV. At 24 hpi cells extracts were loaded and separated in 10% SDS-PAGE, and then analyzed by Western blotting with a rabbit polyclonal antibody against ZIKV prM (Genentex; diluted 1:2,000) or a mouse monoclonal antibody against ZIKV E (BioFront Tech; diluted 1:5,000) to detect the ZIKV prM and E proteins. As loading controls, a rabbit anti-β-actin antibody (Cell Signaling; diluted 1:1,000), and a rabbit anti-VACV E3 antibody (Centro Nacional de Biotecnología; diluted 1:1,000) were used. Anti-mouse or anti-rabbit horseradish peroxidase (HRP)-conjugated antibodies (Sigma; diluted 1:2,000 or 1:5,000, respectively) were used as secondary antibodies. The immune complexes were detected with an HRP-luminol enhanced-chemiluminescence system (ECL Plus, GE Healthcare).

### Genetic stability of MVA-ZIKV

The genetic stability of recombinant MVA-ZIKV was examined as previously described^9,58,63^. MVA-ZIKV (P2 stock) was continuously grown at low MOI in DF-1 cells during 9 passages and then 24 individual plaques were picked from virus derived from passage 9. Next, virus from the 9 passages and the 24 individual plaques from passage 9 were used to infect cells and the expression of ZIKV prM and E proteins was checked by Western Blot as described above.

### Expression of ZIKV E protein from MVA-ZIKV by confocal immunofluorescence microscopy

Immunofluorescence experiments were performed in HeLa cells at 24 hpi, as previously described^9,63^. We used as ER marker a rabbit polyclonal anti-calnexin antibody (BioNova; diluted 1:200) and a mouse monoclonal antibody against ZIKV E protein (BioFront Tech; diluted 1:200) to detect ZIKV E protein in infected permeabilized cells. Anti-ZIKV E and anti-calnexin were detected with mouse secondary antibodies conjugated with the fluorochrome Alexa Fluor 488 (green) and Alexa Fluor 594 (red), respectively (Invitrogen; diluted 1:500). We used DAPI (4′,6′-diamidino-2-phenylindole; Sigma) to stain the cell nuclei. Furthermore, permeabilized and non-permeabilized infected HeLa cells were stained with WGA probe conjugated to the red fluorescent dye Alexa Fluor 594 (Invitrogen; diluted 1:200) and the mouse monoclonal antibody against ZIKV E protein (BioFront Tech; diluted 1:200) to label the cell membrane and the ZIKV E protein, respectively. Images of sections of the cells were taken with a Leica TCS SP5 microscope.

### Detection of VLPs

The detection of VLPs in the supernatants of cells infected with MVA-ZIKV was done as previously described^10,64^. Briefly, HeLa cells were infected with MVA-ZIKV or MVA-WT at an MOI of 10 PFU/cell. Supernatants were collected at 18 hpi, and were concentrated and purified by ultracentrifugation through a 20% sucrose cushion at 25,000 rpm in a Beckmann SW28 rotor for 2 h. Then, the pellet was resuspended in 1 ml of PBS1X, and an aliquot of 50 μl was collected to analyse by western blot the presence of ZIKV M and E proteins. Next, the rest of the sample was loaded into a 20–60% w/v sucrose gradient and ultracentrifugated at 35,000 rpm in a Beckmann SW41 rotor for 18 h. Fractions of 500 μl were taken and the amount of protein and sucrose density was analyzed. The fraction with higher amount of protein (fraction 14) was then used for dialysis through a 0.025-µm-pore-size membrane filter (Merck Millipore) for 12 h. Then, 20 µl of the sample was adsorbed to carbon-coated collodion films mounted on 400-mesh/inch nickel grids (Aname, Spain), and were processed as previously described^10,64^. Samples were incubated with a mouse anti-ZIKV E primary antibody (BioFront Tech; diluted 1:20), an anti-IgG secondary antibody coupled to 10-nm colloidal gold beads (BBInternational; diluted 1:40), and then stained with 2% uranyl acetate (Aname, Spain). Then, pictures were taken using a transmission electron microscope (JEOL JEM-1011; Centro Nacional de Biotecnología, Spain) equipped with a ES1000W Erlangshen charge-coupled-device (CCD) camera (Gatan Inc.) at an acceleration voltage of 40 to 100 kV.

### Mouse immunogenicity study

Female Balb/cOlaHsd mice (6-8-week-old) were purchased from ENVIGO Laboratories and stored in the animal facility of the CNB (Madrid, Spain). A homologous MVA prime/MVA boost immunization protocol was done to evaluate the immunogenicity of the MVA-ZIKV vaccine candidate. Groups of animals (n = 3) were immunized with 1 × 10^7^ PFUs of MVA-WT or MVA-ZIKV by the i.p. route in 200 μl of PBS. Two weeks later, animals received a second dose of 2 × 10^7^ PFUs of MVA-WT or MVA-ZIKV. Ten days post-boost, mice were sacrificed using CO_2_. Blood from of each individual mouse was collected and processed to obtain sera samples to analyze the titers of neutralizing antibodies against ZIKV; and spleens were extracted and processed to measure T cell immune responses to the ZIKV E antigen by an intracellular cytokine staining (ICS) assay. No adverse effects were detected in mice immunized with MVA-ZIKV or MVA-WT.

### Peptides

A ZIKV E peptide pool of the ZIKV PRVABC59 strain (GenPept: AMZ03556) was used in the immunogenicity analysis. Each purified peptide of the ZIKV E peptide pool is at 1 mg per vial, and was obtained through BEI Resources (National Institute of Allergy and Infectious Disease, National Institutes of Health, USA). They spanned the entire ZIKV E protein as consecutive 15-mers overlapping by 12 amino acids.

### ICS assay

The magnitude, polyfunctionality, and phenotype of the ZIKV-specific adaptive T cell responses were analyzed by ICS, as previously described^9,40,65,66^. After spleen processing, splenocytes (depleted of red blood cells) were stimulated with 5 μg/ml of the ZIKV E peptide pool. Then, cells were stained for the surface markers, fixed, permeabilized (Cytofix/Cytoperm kit; BD Biosciences), and stained intracellularly with the appropriate fluorochrome-conjugated antibodies, as previously described^9,40,65,66^.

### Plaque reduction neutralization assay (PRNT)

Titers of neutralizing antibodies against ZIKV present in the sera of immunize mice were determined by a PRNT assay using Vero cells, as previously described^64^. Titers of neutralizing antibodies were expressed as the reciprocal of the serum dilution that inhibited plaque formation by 90% (PRNT_90_), relative to samples incubated with negative control sera.

### IFNAR^−/−^ challenge study

To evaluate the efficacy of MVA-ZIKV, a ZIKV challenge study was performed in a biosafety level 3 laboratory at the CISA-INIA, Madrid, Spain. Groups of female IFNAR^−/−^ mice (n = 10 mice/group; 6-week-old) were immunized with 2 × 10^7^ PFUs of MVA-WT (one dose, at day 0) or MVA-ZIKV (one or two doses, at days 0 and 14, respectively) by the i.p. route. At days 13 and 27 post-prime immunization (13 days after one or two immunizations, respectively) mice were bled from check bleedings and serum samples were used to determine the levels of neutralizing antibodies against ZIKV by a PRNT assay, as described above. No adverse effects were observed in mice immunized with MVA-ZIKV or MVA-WT. Four weeks after the first immunization, mice (at an age of 10 weeks) were challenged with 10^4^ PFUs of ZIKV (PA259459, strain Panama) via the i.p. route. After challenge, blood samples were collected at days 2 (n = 5) or 3 (n = 5), and ZIKV viremia was determined by quantitative RT-PCR and by a plaque assay as described below. Mice were checked daily after challenge for signs of disease and body weight during 15 days. At 15 days post-challenge mice were sacrificed.

### Analysis of ZIKV viremia

Viral RNA was automatically extracted from serum samples using QIAmp® Viral RNA Mini Kit (Qiagen) and a QIAcube apparatus (Qiagen). The amount of viral RNA was determined by real-time fluorogenic RT-PCR in a Rotorgene 3000 equipment (Corbett Research) using the High Scriptools-Quantimix Easy probes kit (Biotools) and a previously described primer set and probe specific for ZIKV^67^. Genomic equivalents to PFU/ml were calculated by comparison with 10-fold serial dilutions of ZIKV RNA extracted from previously titrated samples. The amount of ZIKV infectious particles in serum samples from infected mice was also determined by titration of 10-fold dilutions of serum samples in Vero cells grown in semisolid agarose medium.

### Data analysis and statistical procedures

Statistical analysis of the ICS data was realized as previously described^62,68^, using an approach that corrects measurements for the medium response (RPMI), with calculation of confidence intervals and P values. Only antigen response values significantly larger than the corresponding RPMI values are represented. Background values were subtracted from all values used to allow analysis of proportionate representation of responses. The statistical significance of neutralization measurement (PRNT_90_) in Balb/c sera was determined by an unpaired t-test, and in IFNAR^−/−^ mice by analysis of the variance (ANOVA) applying Bonferroni’s correction for multiple comparisons. The statistical significance of viremia was also determined by ANOVA applying Bonferroni’s correction for multiple comparisons. Statistically significant differences in the figures are denoted by *P < 0.05; **P < 0.005; ***P < 0.001; ****P < 0.0001).

## Electronic supplementary material

Table S1
