# Supplementary material for: A Vaccine Based on a Modified Vaccinia Virus Ankara Vector Expressing Zika Virus Structural Proteins Controls Zika Virus Replication in Mice
Source: Sci Rep. 2018 Nov 26;8:17385. doi: 10.1038/s41598-018-35724-6 (PMC6255889; doi:10.1038/s41598-018-35724-6)
Supplement: Supplementary file 1 — Table S1 [file 41598_2018_35724_MOESM1_ESM.pdf]

A Vaccine Based on a Modified Vaccinia Virus Ankara Vector Expressing Zika Virus Structural Proteins Controls Zika Virus Replication in Mice

Patricia Pérez[a], María Q. Marín[a], Adrián Lázaro-Frías[a], Nereida Jiménez de Oya[b], Ana-Belén Blázquez[b], Estela Escribano-Romero[b], Carlos Óscar S. Sorzano[c], Javier Ortego[d], Juan-Carlos Sáiz[b], Mariano Esteban[a]#, Miguel A. Martín-Acebes[b]# and Juan García-Arriaza[a]#

[a] Department of Molecular and Cellular Biology, Centro Nacional de Biotecnología (CNB), Consejo Superior de Investigaciones Científicas (CSIC), Madrid, Spain.

[b] Department of Biotechnology, Instituto Nacional de Investigación y Tecnología Agraria y Alimentaria (INIA), Madrid, Spain.

[c] Biocomputing Unit, Centro Nacional de Biotecnología (CNB), Consejo Superior de Investigaciones Científicas (CSIC), Madrid, Spain.

[d] Centro de Investigación en Sanidad Animal, INIA-CISA, Valdeolmos, Madrid, Spain.

#Address correspondence to Mariano Esteban (mesteban@cnb.csic.es), Miguel A. Martín-Acebes (martin.mangel@inia.es) and Juan García-Arriaza (jfgarcia@cnb.csic.es)

	Total amount of splenocytes	CD8 (Total number)	CD8 (Total ZIKV-specific response)	CD107+ IFNγ+TNFα+IL-2	CD107+ IFNγ+TNFα	CD107+ IFNγ	CD107+ TNFα	CD107	Total CD107	Total IFNγ	Total IL-2	Total TNFα
MVA-ZIKV / MVA-ZIKV	176,400,000	20,072,141	952,005 (4.74%)	54,934 (0.27%)	258,797 (1.29%)	208,058 (1.04%)	18,200 (0.09%)	412,017 (2.05%)	952,005 (4.74%)	521,789 (2.60%)	54,934 (0.27%)	331,931 (1.65%)
MVA-WT / MVA-WT	236,400,000	26,819,423	0 (0%)	0 (0%)	0 (0%)	0 (0%)	0 (0%)	0 (0%)	0 (0%)	0 (0%)	0 (0%)	0 (0%)

Table S1. Absolute numbers of ZIKV-specific CD8$^+$ T cells induced in mice immunized with MVA-ZIKV or MVA-WT. The values represent the estimated absolute numbers of splenocytes, CD8$^+$ T cells and ZIKV-specific CD8$^+$ T cells producing CD107a and/or IFN-γ and/or TNF-α and/or IL-2 against the ZIKV E protein peptide pool, obtained from Balb/c mice immunized with MVA-ZIKV or MVA-WT. The percentages are included between brackets, and are also represented in Fig. 5.
